# Supplementary material for: A fault-intrusion-tolerant system and deadline-aware algorithm for scheduling scientific workflow in the cloud
Source: PeerJ Comput Sci. 2021 Nov 2;7:e747. doi: 10.7717/peerj-cs.747 (PMC8576554; doi:10.7717/peerj-cs.747)
Supplement: Supplemental Information 6 [file peerj-cs-07-747-s006.zip › Myproject1.1/docs/org/cloudbus/cloudsim/core/FutureQueue.html]

FutureQueue


JavaScript is disabled on your browser.


- Overview
- Package
- Class
- Use
- Tree
- Deprecated
- Index
- Help

- Prev Class
- Next Class

- Frames
- No Frames

- All Classes

- Summary:
- Nested |
- Field |
- Constr |
- Method

- Detail:
- Field |
- Constr |
- Method


org.cloudbus.cloudsim.core

## Class FutureQueue

- java.lang.Object
- - org.cloudbus.cloudsim.core.FutureQueue

- ---

    

  ```
  public class FutureQueue
  extends java.lang.Object
  ```

  This class implements the future event queue used by `Simulation`. The event queue uses a
  `TreeSet` in order to store the events.

  Since:
  :   CloudSim Toolkit 1.0

  See Also:
  :   `Simulation`,
      `TreeSet`

- - ### Constructor Summary

    Constructors

    Constructor and Description

    `FutureQueue()`
  - ### Method Summary

    Methods

    | Modifier and Type | Method and Description |
    | --- | --- |
    | `void` | `addEvent(SimEvent newEvent)` Add a new event to the queue. |
    | `void` | `addEventFirst(SimEvent newEvent)` Add a new event to the head of the queue. |
    | `void` | `clear()` Clears the queue. |
    | `java.util.Iterator<SimEvent>` | `iterator()` Returns an iterator to the queue. |
    | `boolean` | `remove(SimEvent event)` Removes the event from the queue. |
    | `boolean` | `removeAll(java.util.Collection<SimEvent> events)` Removes all the events from the queue. |
    | `int` | `size()` Returns the size of this event queue. |

    - ### Methods inherited from class java.lang.Object

      `clone, equals, finalize, getClass, hashCode, notify, notifyAll, toString, wait, wait, wait`

- - ### Constructor Detail


    - #### FutureQueue

      ```
      public FutureQueue()
      ```
  - ### Method Detail


    - #### addEvent

      ```
      public void addEvent(SimEvent newEvent)
      ```

      Add a new event to the queue. Adding a new event to the queue preserves the temporal order of
      the events in the queue.

      Parameters:
      :   `newEvent` - The event to be put in the queue.


    - #### addEventFirst

      ```
      public void addEventFirst(SimEvent newEvent)
      ```

      Add a new event to the head of the queue.

      Parameters:
      :   `newEvent` - The event to be put in the queue.


    - #### iterator

      ```
      public java.util.Iterator<SimEvent> iterator()
      ```

      Returns an iterator to the queue.

      Returns:
      :   the iterator


    - #### size

      ```
      public int size()
      ```

      Returns the size of this event queue.

      Returns:
      :   the size


    - #### remove

      ```
      public boolean remove(SimEvent event)
      ```

      Removes the event from the queue.

      Parameters:
      :   `event` - the event

      Returns:
      :   true, if successful


    - #### removeAll

      ```
      public boolean removeAll(java.util.Collection<SimEvent> events)
      ```

      Removes all the events from the queue.

      Parameters:
      :   `events` - the events

      Returns:
      :   true, if successful


    - #### clear

      ```
      public void clear()
      ```

      Clears the queue.


- Overview
- Package
- Class
- Use
- Tree
- Deprecated
- Index
- Help

- Prev Class
- Next Class

- Frames
- No Frames

- All Classes

- Summary:
- Nested |
- Field |
- Constr |
- Method

- Detail:
- Field |
- Constr |
- Method
